# Supplementary material for: Role and mechanism of NCAPD3 in promoting malignant behaviors in gastric cancer
Source: Front Pharmacol. 2024 Apr 22;15:1341039. doi: 10.3389/fphar.2024.1341039 (PMC11070777; doi:10.3389/fphar.2024.1341039)
Supplement: Supplementary file 11 [file DataSheet2.ZIP › GSEA/Oncogenic signature/my_analysis.Gsea.1599461962066/LTE2_UP.V1_UP.html]

Details for gene set LTE2\_UP.V1\_UP[GSEA]

|  || Dataset | filtered\_dataset.sample\_info.cls#WT\_versus\_NCAPD3\_MUT |
| Phenotype | sample\_info.cls#WT\_versus\_NCAPD3\_MUT |
| Upregulated in class | NCAPD3\_MUT |
| GeneSet | LTE2\_UP.V1\_UP |
| Enrichment Score (ES) | -0.40187255 |
| Normalized Enrichment Score (NES) | -1.8761927 |
| Nominal p-value | 0.0046620048 |
| FDR q-value | 0.033722296 |
| FWER p-Value | 0.279 |
Table: GSEA Results Summary

  

Fig 1: Enrichment plot: LTE2\_UP.V1\_UP      
 Profile of the Running ES Score & Positions of GeneSet Members on the Rank Ordered List

  

| SYMBOL | TITLE | RANK IN GENE LIST | RANK METRIC SCORE | RUNNING ES | CORE ENRICHMENT || 1 | 79789 | CLMN | 160 | 0.717 | -0.0538 | No |
| 2 | 1601 | DAB2 | 327 | 0.577 | -0.1240 | No |
| 3 | 81031 | SLC2A10 | 539 | 0.453 | -0.2372 | No |
| 4 | 89910 | UBE3B | 693 | 0.381 | -0.3147 | No |
| 5 | 25825 | BACE2 | 756 | 0.344 | -0.3299 | No |
| 6 | 9749 | PHACTR2 | 806 | 0.319 | -0.3379 | No |
| 7 | 6558 | SLC12A2 | 839 | 0.276 | -0.3373 | No |
| 8 | 360 | AQP3 | 857 | -0.263 | -0.3270 | No |
| 9 | 694 | BTG1 | 901 | -0.326 | -0.3301 | No |
| 10 | 51141 | INSIG2 | 903 | -0.328 | -0.3027 | No |
| 11 | 4680 | CEACAM6 | 955 | -0.374 | -0.3074 | No |
| 12 | 10974 | ADIRF | 962 | -0.382 | -0.2790 | No |
| 13 | 11138 | TBC1D8 | 1026 | -0.424 | -0.2881 | No |
| 14 | 665 | BNIP3L | 1185 | -0.556 | -0.3543 | Yes |
| 15 | 64759 | TNS3 | 1197 | -0.572 | -0.3133 | Yes |
| 16 | 26999 | CYFIP2 | 1234 | -0.611 | -0.2869 | Yes |
| 17 | 80328 | ULBP2 | 1277 | -0.664 | -0.2603 | Yes |
| 18 | 57553 | MICAL3 | 1318 | -0.732 | -0.2264 | Yes |
| 19 | 6646 | SOAT1 | 1333 | -0.755 | -0.1719 | Yes |
| 20 | 2650 | GCNT1 | 1342 | -0.781 | -0.1108 | Yes |
| 21 | 26298 | EHF | 1378 | -0.919 | -0.0573 | Yes |
| 22 | 64764 | CREB3L2 | 1382 | -0.930 | 0.0202 | Yes |
Table: GSEA details [plain text format]

  

Fig 2: LTE2\_UP.V1\_UP      
 Blue-Pink O' Gram in the Space of the Analyzed GeneSet

  

Fig 3: LTE2\_UP.V1\_UP: Random ES distribution      
 Gene set null distribution of ES for **LTE2\_UP.V1\_UP**
